# Refined Deep-Sea Water Suppresses Inflammatory Responses via the MAPK/AP-1 and NF-κB Signaling Pathway in LPS-Treated RAW 264.7 Macrophage Cells

**DOI:** 10.3390/ijms18112282

**Published:** 2017-10-31

**Authors:** So-Young Chun, Kyu-Shik Lee, Kyung-Soo Nam

**Affiliations:** Department of Pharmacology, School of Medicine and Intractable Disease Research Center, Dongguk University, Gyeongju 38066, Korea; iaha1234@dongguk.ac.kr (S.-Y.C.); there1@dongguk.ac.kr (K.-S.L.)

**Keywords:** atopic dermatitis, inflammation, refined deep-sea water, lipopolysaccharide, RAW 264.7 macrophage cells

## Abstract

Atopic dermatitis (AD) is a type of inflammatory skin disease caused by genetics, immune system dysfunction, and environmental stresses. It is, however, still considered to be a refractory disease. Macrophages are inflammatory immune cells that infiltrate the skin and induce inflammation. We investigated the effect of refined deep-sea water (RDSW) on lipopolysaccharide (LPS)-induced inflammatory response in RAW 264.7 macrophage cells. The results showed that RDSW suppressed the expressions of inducible nitric oxide synthase (iNOS) and cyclooxygenase (COX)-2. Furthermore, nitric oxide, a product of iNOS, and prostaglandin (PG) D_2_ and PGE_2_, products of COX-2, were significantly inhibited by RDSW in a hardness-dependent manner. Moreover, we found that RDSW reversed the release of histamines and regressed the mRNA expressions and production of pro-inflammatory cytokines, such as tumor necrosis factor-α, interleukin (IL)-1β, IL-6, and IL-10, and vascular endothelial growth factor, in a hardness-dependent manner. We also found that the suppressive effect of RDSW on LPS-induced inflammatory responses was regulated by the inhibition of NF-κB nuclear translocation, and ERK 1/2 and JNK 1/2 mediated the suppression of c-Jun and c-Fos expressions. In conclusion, the present investigation suggests the possibility that RDSW may be used to treat and/or prevent inflammatory diseases, including AD.

## 1. Introduction

Atopic dermatitis (AD) is the most common, chronic, and relapsing inflammatory disease of the skin. It affects approximately 2–10% of adults and 15–30% of children, worldwide [1,2,3,4]. It is characterized by dryness, itchiness, pruritic, and eczematous skin lesions [5,6], and it is known to be caused by pharmacological factors, genetic factors, and/or environmental factors, such as house dust mites, food allergens, and microbial antigens [7,8]. AD is an inflammation of the skin that is accompanied by activated immune cells, especially macrophages. Particularly, high levels of pro-inflammatory cytokines that were released from macrophage, accumulated in inflamed skin, were seen in the blood of AD patients [9]. Macrophages play key roles in the immune system, and the major activating mediator of macrophages is lipopolysaccharide (LPS), an element of the cell wall of Gram-negative bacteria. LPS triggers the toll-like receptor 4 (TLR4), resulting in the transcriptional activation of pro-inflammatory gene expression [10]. The pivotal mediators of the various inflammatory responses, including inducible nitric oxide synthase (iNOS), cyclooxygenase (COX)-2, chemokines, and pro-inflammatory cytokines (tumor necrosis factor (TNF)-α, interleukin (IL)-1β, IL-6, and IL-10) are primarily expressed in macrophages. Moreover, these pro-inflammatory mediators are involved in various acute and chronic inflammatory diseases [11]. The activated macrophages increase the inflammatory responses, including the migration of macrophages, phagocytosis, secretion of cytokines, production of chemical mediators, such as nitric oxide (NO) and prostaglandin (PG)s, and the generation of reactive oxygen species [12,13]. LPS activates two distinct downstream signaling pathways; one, the nuclear factor kappa-light-chain-enhancer of the activated B cells (NF-κB) pathway, and two, the mitogen-activated protein kinases (MAPKs) pathway. Phosphorylated MAPKs participates in the NF-κB activation and gene expression of iNOS [14].

Histamine plays various roles as an autacoid. Among them, it representatively regulates the allergic inflammatory reaction and plays an essential role in acute and chronic inflammation and hypersensitivity. The elevated levels of histamine have been shown to be involved in inflammatory diseases and allergies, such as eczematous skin diseases and lesions of psoriasis. Moreover, histamine facilitates the growth of neoplasia and promotes angiogenesis in inflammatory tissues [15,16]. Angiogenesis, in a chronic inflammatory state, is a normal and vital process in the tissue growth and development, as well as in the migration of inflammatory cells into the inflammatory site. Additionally, angiogenesis is enhanced by NO and PGE_2_, inflammation mediators. Thus, proliferative inflammation and the progression of chronic inflammation depend on angiogenesis [15,17].

Deep-sea water (DSW), defined as sea water acquired from the depth of more than 200 m, has been proven to have numerous advantages, such as high purity, nutrition, stability, and low temperature. DSW contains abundant minerals, such as calcium (Ca), magnesium (Mg), potassium (K), sodium (Na), and zinc (Zn) [18,19]; among these, there are high levels of Mg and Ca. Recently, DSW has been considered as a promising ingredient in food and medical applications due to its efficacy of natural minerals. In previous reports, DSW has been shown to prevent atherosclerosis [20], attenuate the level of plasma lipid and adipogenic protein expression [21], and inhibit visceral fat accumulation and liver steatosis [22]. Furthermore, we previously demonstrated that DSW has anti-cancer metastatic [23], cardioprotective [24], and hypocholesterolemic effects [25,26].

Sugimoto et al., showed that inflammatory cytokine production was regressed by Mg, and some investigations demonstrated the benefits of DSW in atopic eczema/dermatitis syndrome [6,27,28]. These reports imply that Mg plays an important role in the inflammation and atopic eczema; and DSW is a good source of Mg. However, the regulatory mechanism has not been verified. Therefore, we investigated the effects of refined DSW (RDSW) on LPS-induced inflammatory response and its regulatory mechanism in RAW 264.7 macrophage cells.

## 2. Results

### 2.1. Histamine Release was Inhibited by Refined Deep-Sea Water (RDSW) on LPS-Induced RAW 264.7 Macrophage Cells

**Figure 1:**
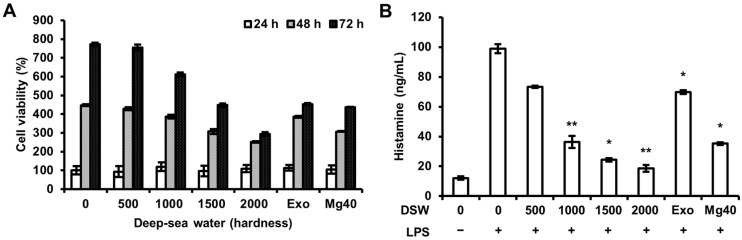
Effect of RDSW on cell viability and histamine release in LPS-treated RAW 264.7 macrophage cells. (**A**) Cells were treated with various hardness (0, 500, 1000, 1500, and 2000) of RDSW for 24, 48, or 72 h, and cell viabilities were assessed by SRB; (**B**) The release of histamine was measured in a conditioned medium. The cells were pre-treated with RDSW for 24 h prior to the stimulation of 1 µg/mL LPS for 24 h. All experiments were performed in triplicates, and data are shown as the mean ± SD. * *p* < 0.05 and ** *p* < 0.01 compared to LPS treatment only. Exo: hardness 1500 of exogenous Mg and Ca mixture, Mg:Ca = 3.5:1; Mg40: hardness 1500 of high Mg DSW, Mg:Ca = 40:1.

To examine the effect of RDSW, we simultaneously evaluated the cell viability at various hardness (0, 500, 1000, 1500, and 2000) of RDSW for 24, 48, or 72 h by the sulforhodamine B (SRB) assay. As shown in Figure 1A, the survival of RAW 264.7 macrophage cells were not affected by an exposure to different hardness of RDSW compared with that of the desalinated DSW (hardness 0). We also evaluated LPS-induced release of histamine in RAW 264.7 macrophage cells (Figure 1B). Contrastingly, highly increased histamine release by LPS was significantly reduced by RDSW in a hardness-dependent manner (Figure 1B).

### 2.2. RDSW Inhibited iNOS Expressions and NO Production in LPS-Stimulated RAW 264.7 Macrophage Cells

**Figure 2:**
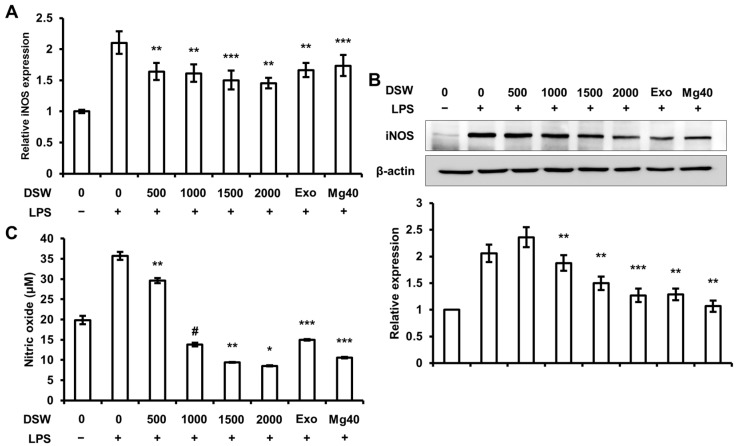
Effect of RDSW on LPS-stimulated iNOS expressions and NO production. Effect of RDSW on LPS-induced (**A**) mRNA and (**B**) protein expressions of iNOS in RAW 264.7 macrophage cells. (**A**) Glyceraldehyde 3-phosphate dehydrogenase (GAPDH) was used as an internal control; (**B**) band density was normalized with β-actin; (**C**) The production of NO was measured in a conditioned medium of cells pre-treated with RDSW for 24 h prior to the stimulation of 1 µg/mL LPS for 24 h. The amount of NO presented as nitrite in the medium was measured by Griess reagent and normalized with the amount of total protein in each sample. All experiments were performed in triplicate, and all data are shown as the mean ± SD. * *p* < 0.05, ** *p* < 0.01, *** *p* < 0.001 and # *p* < 0.0001 compared to the LPS-induced control. Exo: hardness 1500 of exogenous Mg and Ca mixture, Mg:Ca = 3.5:1; Mg40: hardness 1500 of high Mg DSW, Mg:Ca = 40:1.

We investigated the effect of RDSW on LPS-stimulated expression of iNOS, which is one of the pro-inflammatory mediators, and NO production in RAW 264.7 macrophage cells. NO synthesized by iNOS is a prominent mediator of inflammatory diseases [14]. NO production activated by LPS is known to be regulated by MAPKs and NF-κB signaling pathways [11]. The results showed that RDSW inhibited iNOS mRNA (Figure 2A) and protein (Figure 2B) expressions. We also confirmed that there was LPS-induced NO production in RAW 264.7 macrophage cells (Figure 2C). The results showed that RDSW decreased the production of NO induced by LPS, especially at hardness of 1000, 1500, and 2000. Thus, the results show that RDSW substantially reduced the expressions of iNOS and production of NO in LPS-induced RAW 264.7 macrophage cells.

### 2.3. RDSW Also Suppressed COX-2 Expressions, PGD_2_ and PGE_2_ Secretions in LPS-Induced RAW 264.7 Macrophage Cells

**Figure 3:**
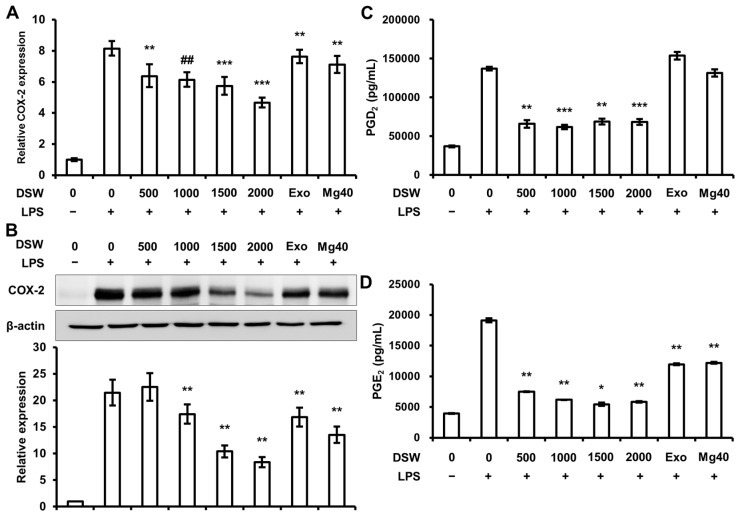
Effect of RDSW on LPS-induced COX-2 expressions and PGD_2_ and PGE_2_ secretions. Effects of RDSW on LPS-induced (**A**) mRNA and (**B**) protein expressions of COX-2 in RAW 264.7 macrophage cells. (**A**) GAPDH was used as an internal control; (**B**) band density was normalized with β-actin. The secretions of (**C**) PGD_2_ and (**D**) PGE_2_ were measured in a conditioned medium pre-treated with RDSW for 24 h prior to the stimulation of 1 µg/mL LPS for 24 h. The amount of PGD_2_ and PGE_2_ secretion in the medium was measured by ELISA kits in accordance with the manufacturer’s instructions and was normalized with the amount of total protein in each sample. All experiments were performed in triplicates, and all data are shown as the mean ± SD. * *p* < 0.05, ** *p* < 0.01, *** *p* < 0.001 and ## *p* < 0.00001 compared to the LPS-induced control. Exo: hardness 1500 of exogenous Mg and Ca mixture, Mg:Ca = 3.5:1; Mg40: hardness 1500 of high Mg DSW, Mg:Ca = 40:1.

To study the effects of RDSW on LPS-induced COX-2 expressions, PGD_2_ and PGE_2_ secretions, the cells were treated and harvested as aforementioned. COX-2, a major pro-inflammatory mediator, was also found to be induced by LPS, resulting in the secretion of PGD_2_ and PGE_2_, which play a role in the allergic and inflammatory responses [12]. As shown in Figure 3A,B, COX-2 mRNA and protein expressions were suppressed by RDSW in RAW 264.7 macrophage cells, in a hardness dependent manner. Moreover, the secretion of PGD_2_ (Figure 3C) and PGE_2_ (Figure 3D) synthesized by COX-2 were significantly decreased by RDSW. Hence, our results indicated that RDSW considerably suppressed the expression of COX-2 and secretion of PGD_2_ and PGE_2_ in LPS-stimulated RAW 264.7 macrophage cells.

### 2.4. Production and Expression of Pro-Inflammatory Cytokines and VEGF Expression were Abolished by RDSW in LPS-Induced RAW 264.7 Macrophage Cells

**Figure 4:**
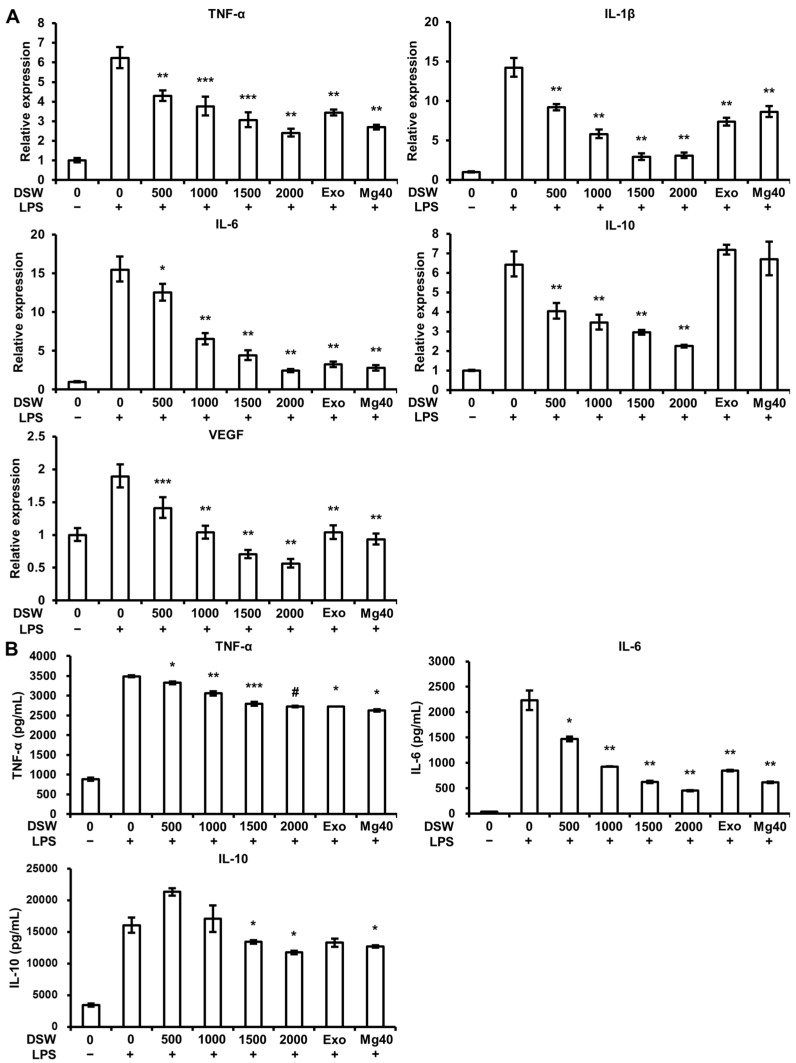
Effect of RDSW on LPS-induced pro-inflammatory cytokines expression and production, and VEGF expression. (**A**) The inhibitory effect of RDSW on TNF-α, IL-1β, IL-6, IL-10, and VEGF mRNA expressions. GAPDH was used as an internal control for mRNA expressions. (**B**) The inhibition of the production of pro-inflammatory cytokines (TNF-α, IL-6, and IL-10) by RDSW. The amount of pro-inflammatory cytokines in the conditioned medium was measured by ELISA kits according to the manufacturer’s instructions and was normalized with the amount of total protein in each sample. All experiments were performed in triplicates, and all data are shown as the mean ± SD. * *p* < 0.05, ** *p* < 0.01, *** *p* < 0.001 and # *p* < 0.0001 compared to the LPS-induced control. Exo: hardness 1500 of exogenous Mg and Ca mixture, Mg:Ca = 3.5:1; Mg40: hardness 1500 of high Mg DSW, Mg:Ca = 40:1.

The levels of various inflammatory mediators, including pro-inflammatory cytokines, such as TNF-α, IL-1β, IL-6, and IL-10, and vascular endothelial growth factor (VEGF), one of the angiogenic factors, are high in the skin of AD patients. We assessed the effect of RDSW on LPS-induced TNF-α, IL-1β, IL-6, and IL-10 transcriptions and productions, as well as on the VEGF gene expression in RAW 264.7 macrophage cells. As shown in Figure 4A, RDSW dramatically lessened the pro-inflammatory cytokines (TNF-α, IL-1β, IL-6, and IL-10) and VEGF expressions in a hardness-dependent manner. Furthermore, increased productions of TNF-α and IL-6 by LPS were hardness-dependently reversed by RDSW, and the production of IL-10 was inhibited by RDSW with hardnesses of 1500 and 2000 (Figure 4B).

### 2.5. RDSW Diminished LPS-Stimulated IκB Phosphorylation, NF-κB Nuclear Translocation, and Activations of ERK 1/2 and JNK 1/2 in RAW 264.7 Macrophage Cells

**Figure 5:**
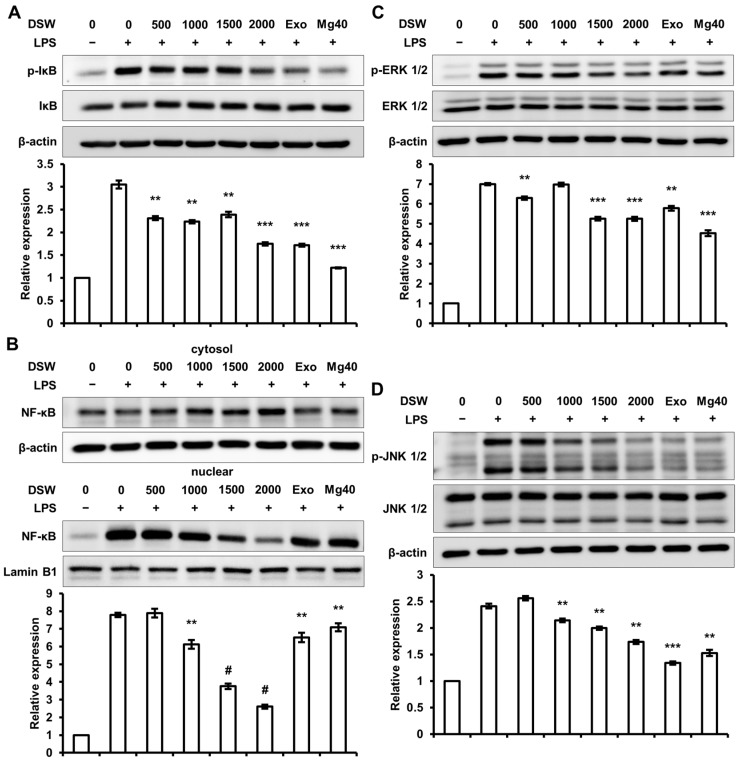
Effect of RDSW on phosphorylation of IκB, nuclear translocation of NF-κB, and activations of ERK 1/2 and JNK 1/2 in LPS-induced RAW 264.7 macrophage cells. The cells were pre-treated with RDSW for 24 h prior to the stimulation of LPS (1 µg/mL) for various incubation times (IκB; 15 min, NF-κB, ERK 1/2 and JNK 1/2; 30 min). RDSW inhibited LPS-induced (**A**) phosphorylation of IκB, (**B**) nuclear translocation of NF-κB, and activations of (**C**) ERK 1/2 and (**D**) JNK 1/2. β-actin was used as the internal control for the whole cell lysate and cytosolic fraction, and Lamin B1 was used as the internal control for the nuclear fraction. Band densities of p-IκB, NF-κB, p-ERK 1/2, and p-JNK 1/2 were normalized with IκB, Lamin B1, ERK 1/2 and JNK 1/2, respectively. All experiments were performed in triplicate, and all data are shown as the mean ± SD. ** *p* < 0.01, *** *p* < 0.001 and # *p* < 0.0001 compared to the LPS-induced control. Exo: hardness 1500 of exogenous Mg and Ca mixture, Mg:Ca = 3.5:1; Mg40: hardness 1500 of high Mg DSW, Mg:Ca = 40:1.

To study the regulatory signaling pathway of RDSW on inflammatory response, we evaluated the effect of RDSW on LPS-stimulated nuclear factor of kappa light polypeptide gene enhancer in B cells inhibitor (IκB) phosphorylation, NF-κB nuclear translocation, and activations of extracellular signal-regulated kinases (ERK) 1/2 and c-Jun N-terminal kinases (JNK) 1/2 in RAW 264.7 macrophage cells. As shown in Figure 5A, the phosphorylation of IκB was decreased by RDSW, and the nuclear translocation of NF-κB was also inhibited by RDSW in a hardness-dependent manner (Figure 5B). Moreover, as can be seen from Figure 5C,D, both ERK 1/2 and JNK 1/2 phosphorylations were also significantly diminished by RDSW.

### 2.6. Inhibition of c-Jun and c-Fos Expressions by RDSW was Regulated by ERK 1/2 and JNK 1/2 Signaling Pathway

**Figure 6:**
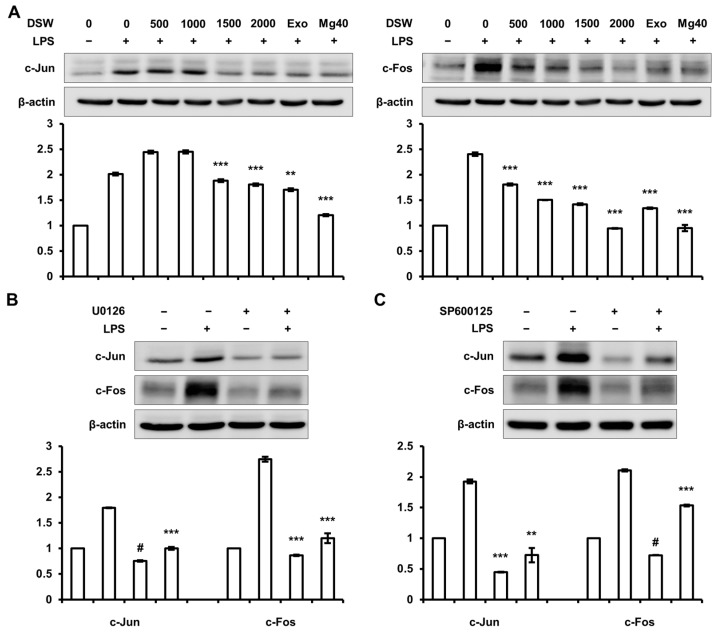
Inhibitory effects of RDSW on LPS-induced inflammatory responses were mediated through the inhibition of the ERK 1/2 and JNK 1/2 signaling pathways. The inhibitory effects of RDSW on LPS-induced (**A**) c-Jun and c-Fos expressions. The involvements of ERK 1/2 and JNK 1/2 signaling pathways in LPS-induced inflammatory response were confirmed by blocking each pathway with a specific inhibitor. The effects of (**B**) 10 μM U0126 (a MEK 1/2 inhibitor) or (**C**) 10 μM SP600125 (a JNK inhibitor) on the expressions of c-Jun and c-Fos in LPS-treated RAW 264.7 macrophage cells. The cells were pre-treated with RDSW for 22 h, and then treated with 10 μM U0126 (a MEK 1/2 inhibitor) or 10 μM SP600125 (a JNK inhibitor) for 2 h prior to LPS stimulation for 1 h. Band density of each sample was normalized with β-actin. All experiments were performed in triplicates, and all data are shown as the mean ± SD. ** *p* < 0.01, *** *p* < 0.001 and # *p* < 0.0001 compared to the LPS-induced control. Exo: hardness 1500 of exogenous Mg and Ca mixture, Mg:Ca = 3.5:1; Mg40: hardness 1500 of high Mg DSW, Mg:Ca = 40:1.

RDSW regressed the LPS-induced ERK 1/2 and JNK 1/2 phosphorylations in RAW 264.7 macrophage cells. Therefore, we confirmed the effect of RDSW on c-Jun and c-Fos, which were AP-1 subunit and regulated by MAPKs, involving ERK 1/2 and JNK 1/2. The result showed that LPS-induced c-Jun and c-Fos expressions were efficiently decreased by RDSW (Figure 6A). Based on this result, we further analyzed whether the inhibitory effect of RDSW on these expressions subsequently affected the ERK 1/2 and JNK 1/2 signaling pathways. Therefore, we blocked the ERK 1/2 and JNK 1/2 signaling pathways using U0126 (a MEK 1/2 inhibitor) and SP600125 (a JNK inhibitor), respectively. The result showed that LPS-induced c-Jun and c-Fos expressions were suppressed by both inhibitors (U0126 and SP600125) (Figure 6B,C). However, both inhibitors had no effect on NF-κB nuclear translocation (data not shown). Consequently, the anti-inflammatory effect of RDSW was mediated by MAPK/AP-1 signaling pathway and regulation of NF-κB nuclear translocation, respectively.

## 3. Discussion

AD is an extremely pruritic and chronic inflammatory skin disease with increasing frequency. Although some reagents, such as steroids, anti-histamines, and immunosuppressants, are applicable in treating AD, they have many side effects and are restricted in their efficacy for long-time use. Recently, the development of alternative and compensatory therapies or applications of enriched minerals for AD treatment have been reported [5,6,9].

Macrophages are crucial immune cells that mediate allergic inflammatory responses. It is known that the gene expression of various inflammatory mediators can be induced by external antigen stimulators, such as LPS. LPS stimulates TLR4, inducing pro-inflammatory cytokines, including IL-1β, IL-6, IL-12, and TNF-α in macrophages and dendritic cells [29]. As shown in Figure 4, RDSW inhibited the gene expression and secretion of pro-inflammatory cytokines in LPS-treated RAW 264.7 macrophage cells. The results describe that RDSW should prevent the development of inflammation mediated by macrophages.

iNOS is a major producer of NO and plays vital roles in patients with inflammatory diseases, including AD, rhinitis, and pollinosis, when it is stimulated by bacterial lipopolysaccharides or various cytokines, such as IL-1β, IL-2, and TNF-α. Moreover, the COX-2 expression is evaluated in inflammation-related cells in response to the stimulation with cytokines during the immune reaction, resulting in the secretion of PGD_2_ and PGE_2_ [5,30]. PGD_2_ is a known mediator for inflammatory pathological or physiological stimuli [12,31], and PGE_2_ is directly transported into the tissues, stimulating inflammation [32]. Elevated levels of PGE_2_ are detected in the inflammatory lesions, such as arthritic joints [32]. Furthermore, PGE_2_ enhances angiogenesis. VEGF is a crucial angiogenic mediator. Inflammatory cells participate in the regulation of VEGF expression via the secretion of cytokines, such as TNF-α, TGF-β, IL-1, IL-6, IL-8, and IL-18, which enhance angiogenesis through a direct regulation or induction of VEGF [33]. Our investigation showed a suppression of inflammatory mediators, including NO, PGD_2_, and PGE_2_, with down-regulation of iNOS and COX-2 expressions in LPS-treated RAW 264.7 macrophage cells (Figure 2 and Figure 3). These results support the relationship between pro-inflammatory cytokines and VEGF gene expression, as well as PGD_2_ and PGE_2_ production. Moreover, the results demonstrate that RDSW reduces the inflammation activity by attenuating the iNOS and COX-2 expressions in macrophages.

An activation of macrophage response to LPS is linked with the phosphorylation of the MAPKs family, such as ERK, JNK, and p38. These kinases directly control the downstream targets comprising transcriptional regulators, Ets-1, Elk/TCF, and AP-1 [34]. Furthermore, the expression of inflammatory cytokines and pro-inflammatory mediators, including iNOS, COX-2, and TNF-α, are also regulated by NF-κB [14,35]. Here, we showed that LPS induced NF-κB nuclear translocation, as well as ERK 1/2 and JNK 1/2 phosphorylations and upregulated the expressions of c-Jun and c-Fos (Figure 5). Moreover, RDSW reversed the LPS-enhanced NF-κB nuclear translocation, ERK 1/2 and JNK 1/2 phosphorylations, and c-Jun and c-Fos expressions (Figure 5). The results demonstrate the importance of ERK 1/2, JNK 1/2, and NF-κB in LPS-induced inflammatory response; and that anti-inflammatory activity of RDSW is mediated by the MAPKs and NF-κB signaling pathways.

DSW is abundant in minerals, particularly Mg and Ca. Mg is important for many physiological processes, such as enzyme functions and energy metabolism in the body, and Ca has various advantages, including bone development and density [36]. Therefore, they are crucial minerals for humans. The anti-inflammatory and wound-healing traits of Mg are also being recognized. James et al., reported that the application of an aluminum magnesium hydroxide stearate-based skin barrier protection cream, used to manage eczematous dermatitis, restore skin moisture and skin lipid replacement, improving dermatitis symptoms [37]. Proksch et al., reported that by bathing with magnesium-rich Dead Sea salt solution, skin hydration is improved and inflammation in atopic dry skin is reduced [38]. Furthermore, Nourbakhsh et al., showed that Mg 2% combined with Calendula cream in children aged less than two years was a good treatment for diaper dermatitis, suggesting its use-case for other types of dermatitis [39]. Moreover, Hataguchi et al., demonstrated that drinking DSW recovered the mineral imbalance with lessening atopic skin lesions [28]. This present investigation revealed that RDSW reversed LPS-induced inflammatory response in RAW 264.7 macrophage cells. This result supports the importance of Mg in an allergic inflammation. In addition, we confirmed the effects of not only RDSW, but also Exo (exogenous Mg and Ca, Mg:Ca = 3.5:1) and Mg40 (high Mg DSW, Mg:Ca = 40:1). We found that the effects of RDSW and Mg40 were better than Exo, suggesting that trace minerals in RDSW may also play some roles in anti-inflammatory activity.

**Figure 7:**
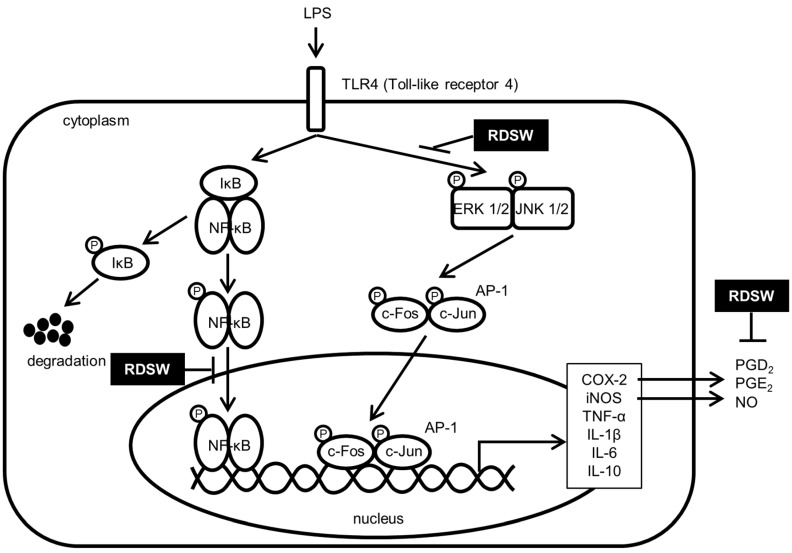
The proposed action mechanism responsible for the suppressive effects of RDSW on the LPS-induced inflammatory signaling pathway.

In summary, the effect of RDSW on the inhibition of LPS-induced inflammatory response in RAW 264.7 macrophage cells was investigated. The results showed that the inhibitory effects of RDSW on histamine release, iNOS and COX-2 expressions, as well as on NO, PGD_2_, and PGE_2_ production, were strengthened. Furthermore, pro-inflammatory cytokines, such as TNF-α, IL-1β, IL-6, and IL-10, production and expression, as well as VEGF expression, were also abolished by RDSW in LPS-treated RAW 264.7 macrophage cells. The results suggest that down-regulations of inflammatory response are mediated by the MAPK/AP-1 and NF-κB signaling pathway (Figure 7). Taken together, the present study suggests that RDSW may be applicable in the treatment for and/or the prevention of inflammatory diseases, including atopic dermatitis.

## 4. Materials and Methods

### 4.1. Cell Culture

RAW 264.7 murine macrophage cells were purchased from the Korean Cell Line Bank (Seoul, Korea). The cells were cultured in Dulbecco’s Modified Eagle Medium (DMEM; Welgene, Daegu, Korea), containing 10% fetal bovine serum (FBS; ATCC, Rockville, MD, USA) and 1% antibiotic-antimycotic solution (Welgene) at 37 °C in a 5% CO_2_ incubator.

### 4.2. Preparation of RDSW

DSW was supplied by the Marine Deep Ocean Water Application Research Center at the Korean Institute of Ocean Science and Technology (Goseong, Gangwon-Do, Korea). DSW was taken 6.7 km off of the Goseong (Gangwon-Do, Korea) coast, at a depth of 500 m. The samples were microfiltered, subjected to reverse osmosis, and concentrated by electrodialysis to obtain desalinated water (hardness 0) and DSW of hardness 3428. The ratio of magnesium-to-calcium presented in DSW (containing 711.72 mg/L Mg, 203.84 mg/L Ca, 39.65 mg/L Na and 17.31 mg/L K) was approximately 3.5:1. It was designated as RDSW. To prepare RDSW containing media, DMEM powder (Sigma, St. Louis, MO, USA) was dissolved in hardness 3428 RDSW and diluted with desalinated RDSW (hardness 0) to obtain hardness 3000 RDSW media. Further serial dilutions were performed to achieve hardnesses of 500, 1000, 1500, and 2000 from 3000 hardness RDSW, using desalinated media (hardness 0). We also prepared exogenous Mg and Ca, its final hardness was 1500, and the ratio of Mg:Ca was 3.5:1 (Exo). Moreover, high-Mg DSW at hardness of 5421 (Mg:Ca = 40:1), containing 1298.26 mg/L Mg, 29.93 mg/L Ca, 376.60 mg/L Na, and 423.20 mg/L K, was obtained by dissolving the extracted minerals in desalinated DSW (hardness 0); its final hardness was 1500, and the ratio of Mg:Ca was 40:1 (Mg40). The hardness was calculated using the following equation:

Hardness of RDSW (mg/L) = Mg (mg/L) × 4.1 + Ca (mg/L) × 2.5.

### 4.3. Cell Treatment

RAW 264.7 macrophage cells were seeded into 6- or 96-well plates and attached for 24 h. Then, the cells were pre-treated with RDSW of various hardness (500, 1000, 1500, and 2000) containing 5% FBS. After 24 h, the cells were treated with 1 μg/mL LPS (Sigma) and further cultured for various time intervals. The cells were harvested for RNA isolation or the preparation of protein lysates. To inhibit the ERK 1/2 or JNK 1/2 signaling pathways, the cells were pre-treated with RDSW for 22 h, and then treated with 10 μM U0126 (a MEK 1/2 inhibitor; LC Laboratories, Woburn, MA, USA) or 10 μM SP600125 (a JNK inhibitor; LC Laboratories) for 2 h prior to 1 μg/mL LPS stimulation for 1 h.

### 4.4. Cell Viability Assay

To evaluate the effect of RDSW on cell viability, we performed SRB assay (Sigma). RAW 264.7 macrophage cells were seeded in 96-well plates at 37 °C and incubated for 24 h. To assess the cell proliferation of RDSW, the cells were treated with RDSW and cultured for an additional 24, 48, or 72 h. To conduct SRB assay, RAW 264.7 macrophage cells were fixed with ice-cold 20% trichloroacetic acid for 1 h at 4 °C. The cells were washed with slow running tap water and then air-dried. The cells were then stained by a 0.4% SRB solution for 30 min at room temperature, and then washed with 1% acetic acid to remove any unbound SRB. The SRB bound to the cells was solubilized with 10 mM Tris-base (pH 10.5), and then the absorbance of SRB was measured at 510 nm using a multi-detection microplate reader (Molecular Devices, Sunnyvale, CA, USA).

### 4.5. Measurement of NO Production

NO production was assessed by measuring the nitrite in the supernatants of cultured RAW 264.7 macrophage cells. The cells were cultured in 96-well plates (3 × 10^4^ cells/well) and pre-treated with RDSW for 24 h before 1 μg/mL LPS stimulation for 24 h. The supernatant was mixed with the Griess reagent and incubated at room temperature for 5 min. The concentration of nitrite was measured by reading at 570 nm using a multi-detection microplate reader.

### 4.6. Measurement of Histamine Release, PGD_2_, PGE_2_, and Various Cytokines’ Production

The cells were grown and treated in 96-well plates as described above. The supernatant was used to determine the histamine release (IBL international GmbH, Hamburg, Germany), PGD_2_ (Cayman Chemical, Ann Arbor, MI, USA), PGE_2_ (Enzo Life Sciences Inc., Farmingdale, NY, USA), TNF-α, IL-1β, IL-6, and IL-10 (BioLegend, San Diego, CA, USA) levels, using the enzyme-linked immunosorbent assay (ELISA) kit in accordance with the manufacturer’s instructions.

### 4.7. RNA Isolation and Quantitative Real-Time Reverse Transcriptase-Polymerase Chain Reaction (qRT-PCR)

The cells were grown and treated in 6-well plates as aforementioned. The total RNA was extracted using the easy-BLUE^TM^ Total RNA Extraction kit (iNtRON Biotechnology Inc., Sungnam, Korea) in accordance with the manufacturer’s instruction. The cDNA was synthesized with Goscript^TM^ Reverse Transcriptase (Promega, Madison, WI, USA). Real-time PCR reactions were performed with QGreen 2X SybrGreen Master Mix (Cellsafe, Suwon, Korea) using Eco^TM^ Real-time PCR (Illumina, San Diego, CA, USA) and products were verified by melting curve analysis. Relative expression levels compared with the control were automatically evaluated by Eco Software v3.1.7 (Illumina). GAPDH was used as an internal control. Primer sequences of the target genes are presented in Table 1.

### 4.8. Western Blotting

The cells were grown and treated in 6-well plates as described above. The cells were lysed with RIPA buffer (150 mM NaCl, 1% Triton X-100, 1% sodium deoxycholate, 0.1% SDS, 50 mM Tris-HCl pH 7.5, and 2 mM ethylenediaminetetraacetic acid), including phosphatase and protease inhibitor cocktail (GenDEPOT, Barker, TX, USA). The lysed cells were then centrifuged at 13,000 rpm for 20 min to collect the whole cell lysate. Protein concentrations were measured with bicinchoninic acid reagent (Sigma). Then, the whole cell lysates were subjected to SDS-polyacrylamide gel electrophoresis and transferred to the polyvinylidene fluoride membranes (Pall Life Sciences, Ann Arbor, MI, USA) at 100 V for 50 min. The membranes were blocked in 5% skim milk in TBS-Tween (50 mM Tris-HCl, 150 mM NaCl, 0.1% Tween 20) for 2 h at room temperature and incubated with the following primary antibodies overnight at 4 °C: iNOS, COX-2, phospho-ERK 1/2, ERK 1/2, phospho-JNK 1/2, JNK 1/2, phospho-NF-κB, NF-κB, phospho-IκB, IκB, c-Jun, c-Fos, and Lamin B1 (Cell Signaling Inc., Beverly, MA, USA), and β-actin (Santa Cruz Biotechnology Inc., Santa Cruz, CA, USA). The blots were then incubated with HRP-conjugated secondary anti-rabbit or anti-mouse antibody (Thermo Fischer Scientific, Waltham, MA, USA) diluted 1:5000 for 1 h at room temperature and developed by a Luminescent Image Analyzer LAS-4000 (Fujifilm, Tokyo, Japan).

### 4.9. Nuclear Fractionation

The cells were washed with ice-cold PBS, and a hypotonic buffer (20 mM Tris-HCl pH 7.4, 10 mM NaCl and 3 mM MgCl_2_) containing phosphatase and protease inhibitor cocktail was added to each sample. The cells were scraped with a policeman scraper and held on ice for 15 min. Then 1/8 volume 10% NP-40 was added, vortexed for 10 s at the highest setting. After incubating on ice for 10 min, the cells were centrifuged at 3000 rpm for 10 min at 4 °C. The supernatant was collected as the cytosolic fraction and the pellet was lysed with a Cell Extraction Buffer (Invitrogen, Carlsbad, CA, USA) containing phosphatase and protease inhibitor cocktail for 30 min on ice. The lysates were centrifuged at 14,000× *g* for 30 min at 4 °C; the collected supernatants were used as the nuclear fraction.

### 4.10. Statistical Analysis

Analysis was conducted using Student’s *t*-test. All experiments were conducted in triplicate, and the results are presented as the mean ± SD. *p* values of <0.05 were considered statistically significant.

## Figures and Tables

**Table 1:** Sequences of primers used for semi-quantitative RT-PCR.

Primers	Forward	Reverse	Product Size (bp)
COX-2	5′-CCTGCTGCCCGACACCTTCA-3′	5′-AGCAACCCGGCCAGCAATCT-3′	139
iNOS	5′-CCTCCTCCACCCTACCAAGT-3′	5′-CACCCAAAGTGCTTCAGTCA-3′	119
TNF-α	5′-ATAGCTCCCAGAAAAGCAAGC-3′	5′-CACCCCGAAGTTCAGTAGACA-3′	258
IL-1β	5′-GCCTTGGGCCTCAAAGGAAAGAATC-3′	5′-GGAAGACACCGATTCCATGGTGAAG-3′	282
IL-6	5′-TGGAGTCACAGAAGGAGTGGCTAAG-3′	5′-TCTGACCACAGTGAGGAATGTCCAC-3′	155
IL-10	5′-CCCTTTGCTATGGTGTCCTT-3′	5′-TGGTTTCTCTTCCCAAGACC-3′	97
VEGF	5′-GTACCTCCACCATGCCAAGT-3′	5′-GCATTCACATCTGCTGTGCT-3′	340
GAPDH	5′-GTATGACTCCACTCACGGCAAA-3′	5′-GGTCTCGCTCCTGGAAGATG-3′	101
